# Practical Surgery

**Published:** 1885-12

**Authors:** 


**Practical Surgery.** By J. EWING MEARS, M. D., with four hundred and ninety illustrations. Second edition. Published by P. Blakiston, Sons & Co.; pp. 795; 12 mo. Price: Cloth, $3.75; sheep, $4.75.

This book, by Dr. Mears, lecturer on surgery in Jefferson Medical College, is really a practical work. The first edition, published in 1878, has been exhausted, and has been received with great favor by the profession, especially by medical students. In fact, we do not know of any other work which would be of greater value to the student for reading in connection with his lectures in this department. The first edition has been carefully revised and enlarged by much new matter, discussing the subjects in the light of the most recent discoveries in antiseptic surgery.

The work of the publishers is exceptionally good.

